# Dissociation between Active and Observational Learning from Positive and Negative Feedback in Parkinsonism

**DOI:** 10.1371/journal.pone.0050250

**Published:** 2012-11-21

**Authors:** Stefan Kobza, Stefano Ferrea, Alfons Schnitzler, Bettina Pollok, Martin Südmeyer, Christian Bellebaum

**Affiliations:** 1 Institute of Cognitive Neuroscience, Department of Neuropsychology, Faculty of Psychology, Ruhr University Bochum, Bochum, Germany; 2 Department of Neurology, Medical Faculty, Heinrich Heine University, Düsseldorf, Germany; 3 Institute of Clinical Neuroscience and Medical Psychology, Heinrich Heine University, Düsseldorf, Germany; University Of São Paulo, Brazil

## Abstract

Feedback to both actively performed and observed behaviour allows adaptation of future actions. Positive feedback leads to increased activity of dopamine neurons in the substantia nigra, whereas dopamine neuron activity is decreased following negative feedback. Dopamine level reduction in unmedicated Parkinson’s Disease patients has been shown to lead to a negative learning bias, i.e. enhanced learning from negative feedback. Recent findings suggest that the neural mechanisms of active and observational learning from feedback might differ, with the striatum playing a less prominent role in observational learning. Therefore, it was hypothesized that unmedicated Parkinson’s Disease patients would show a negative learning bias only in active but not in observational learning. In a between-group design, 19 Parkinson’s Disease patients and 40 healthy controls engaged in either an active or an observational probabilistic feedback-learning task. For both tasks, transfer phases aimed to assess the bias to learn better from positive or negative feedback. As expected, actively learning patients showed a negative learning bias, whereas controls learned better from positive feedback. In contrast, no difference between patients and controls emerged for observational learning, with both groups showing better learning from positive feedback. These findings add to neural models of reinforcement-learning by suggesting that dopamine-modulated input to the striatum plays a minor role in observational learning from feedback. Future research will have to elucidate the specific neural underpinnings of observational learning.

## Introduction

Humans are constantly confronted with situations in which a choice between different actions is required. Decisions can be optimised by learning from the consequences of the chosen actions. Positive consequences, such as reward or positive feedback, increase the frequency of behaviour, whereas actions leading to negative consequences, e.g. punishment or negative feedback, are less likely to reoccur. The dopamine (DA) system plays a crucial role in the processing of rewarding stimuli, as has been shown extensively in studies on monkeys (e.g. [1]–[3]). Activity of DA neurons in the monkey midbrain increases when the monkey receives an unexpected reward, whereas activity decreases when an expected reward is not delivered, thereby reflecting a reward prediction error [4]. A qualitatively similar firing pattern was seen for monetary rewards in single DA neurons of PD patients undergoing surgery for deep brain stimulation [5]. As shown in primate studies, DA neurons of the substantia nigra (SN) are connected with the striatum [6] and the frontal cortex [7]. Similarly in humans, diffusion tensor imaging revealed connections from the SN to the striatum as well as connections between striatum and frontal cortex [8]. Accordingly, functional neuroimaging studies in humans consistently revealed reward-related activations in the striatum and the anterior cingulate cortex (ACC) (e.g. [9]–[11], for reviews see [12], [13]), which – together with the dopaminergic midbrain – constitute the so-called reward system. In particular, the ventral striatum including the nucleus accumbens has been shown to code a reward prediction error during the processing of reward stimuli (e.g. [11], [14]–[16]).

To adapt behaviour based on positive or negative feedback, however, a link between a particular action and the resulting consequences needs to be established. Action-outcome associations can be learned either by active responding or by observing behaviour and the accompanying consequences in other individuals [16]–[21]. Studies examining the processing of positive and negative behavioural outcomes by means of functional magnetic resonance imaging (fMRI) or event-related potentials (ERPs) have suggested that the neural activations related to outcome stimuli depend on whether subjects perceived feedback as dependent on their own behaviour or not. Stronger reward system activations were found for direct action-outcome dependencies compared to situations not involving an action at all [11], [22] or compared to observational learning, where the outcome depends on the behaviour of the observed subject and no active responding by the learner is required [16], [18], [21], [23]. These findings suggest that the neural mechanisms involved in active and observational feedback learning might differ.

Frank and colleagues developed a neural network model which specifies the role of DA neurons and the striatum in active feedback-based learning [24], [25]. DA is thought to dynamically modulate activity in two distinct striatal neuron populations. “Go” cells activated by a burst of DA, e.g. when reward is given, disinhibit the thalamus via the globus pallidus and thereby facilitate action selection by the frontal cortex. In case of reduced DA release, e.g. following punishment, activation of “NoGo” cells leads to increased inhibition of the thalamus, which suppresses actions. As can be concluded from this model, performance in feedback-learning tasks is affected by altered DA levels as e.g. caused by Parkinson’s Disease (PD). In unmedicated PD patients, in whom dopaminergic input from the SN to the striatum is reduced due to massive depletion of DA neurons in the SN [26], [27], Frank and colleagues [24] observed a bias to learn more from negative than positive feedback. In medicated PD patients, however, the reverse pattern was found: PD patients ON medication showed better learning from positive than negative feedback [24], [25].

While imaging and electrophysiological studies suggest that brain structures involved in active learning may differ from those involved in observational learning from feedback (see above), behavioural evidence from brain damaged patients is of high importance but still missing. With respect to one’s own actions, increased and decreased DA levels – as in medicated and unmedicated PD patients – induce relatively enhanced learning from positive and negative feedback, respectively [24], [25]. As was outlined above, no direct link between one’s own action and the outcome is established in observational learning. In the present study it was therefore hypothesized that DA level alterations in PD would affect the learning tendency in active, but not observational learning from feedback. In particular, DA level depletions were expected to lead to a negative bias in active learning, replicating the findings of Frank and colleagues [24], while for observational learning no learning tendency or even a moderate positive bias were expected given recent findings on a positivity effect in observational feedback learning in healthy subjects older than 50 years of age [20]. To this end, PD patients OFF medication were examined either with an active or an observational variant of the probabilistic selection task described by Frank and colleagues (see also [20], [24]), and the (positive or negative) learning tendencies were compared between active and observational learning. For active learning, PD patients showed a negative learning bias relative to controls, whereas patients and controls both learned better from positive feedback in observational learning.

## Materials and Methods

### Ethics Statement

The ethics committee of the Medical Faculty of the Heinrich-Heine-University Düsseldorf, Germany, approved the study (study no. 2849), which conforms to the Declaration of Helsinki.

### Participants

A series of nineteen PD patients attending the Movement Disorder Center of the University Hospital Düsseldorf were prospectively recruited between March 2011 and December 2011 from an ongoing long-term follow-up study. Diagnosis of PD was made according to the UK Brain Bank criteria [28]. For all patients, testing took place in the context of regular follow-up examinations in the hospital. To compare patients’ learning biases between active and observational learning, the 19 PD patients as well as 40 healthy volunteers, who were recruited as control subjects, were randomly assigned to one of two learning groups: 10 patients and 20 controls engaged in an active learning task and the remaining 9 patients and 20 controls learned by observation (see below for details of the tasks). All subjects had normal or corrected-to-normal vision. Mean age and years of education were comparable between the patients and controls and between the two patient and control groups, respectively. Disease duration and long-term follow-up were also comparable between the two patient groups (for demographics, see Table 1). Testing was conducted OFF medication (see Testing procedure for details). The following exclusion criteria were applied for the patients: history of psychiatric disease (e.g. schizophrenia and mania), dementia, advanced PD symptoms (Hoehn and Yahr stage IV or V), documented or suspected history of drug abuse and/or alcoholism, (additional) regular medication affecting the central nervous system, clinical or diagnostic signs of symptomatic or atypical Parkinsonism, and unstable dopaminergic medication within the last two months. The degree of depressive symptoms in the patients was controlled with the German version of the Beck Depression Inventory (BDI-II) [29]. PD patients who were assigned to the active learning task had mean BDI scores of 7.7 (standard deviation [*SD*] = 4.7), and the patients who learned by observation had a mean score of 11.3 (*SD* = 6.2). Higher scores in single patients were at least partially caused by BDI items which ask for motor activity related symptoms. As disturbances in movement control are the core symptoms in PD, higher scores on such items do not necessarily indicate depressed mood (for a review, see [30]). Importantly, the scores for the two groups of patients did not differ significantly (*p* = .16). For the control participants, a history of psychiatric or neurological disorders as well as regular medication affecting the central nervous system and documented or suspected history of drug abuse and/or alcoholism led to exclusion from the study.

**Table 1:** Demographic variables of PD patients and healthy controls.

Group (N)	Age (yrs)	Age range(yrs)	Sex ratio (m:f)	Education (yrs)	UPDRS-III(OFF)	Disease duration (yrs)	Long-term follow-up (months)
PD obs (9)	55.3±9.7	41–74	6∶3	11.1±1.9	24.6±7.4	4.9±2.5	35.3±26.0
PD act (10)	55.8±10.3	36–67	5∶5	10.2±2.1	20.8±4.4	3.9±2.1	26.1±24.8
Controls obs (20)	54.4±11.2	33–76	11∶9	11.4±2.0	N/A	N/A	N/A
Controls act (20)	54.1±10.4	37–71	9∶11	11.2±1.9	N/A	N/A	N/A

obs = observational learners; act = active learners; values indicate mean±SD.

### The Learning Tasks

Two feedback learning tasks were used in the present study: One in which subjects learned actively from their choices and the accompanying outcomes, and one in which subjects observed the choices and outcomes of another person. Both tasks are based on the probabilistic selection task introduced by Frank and colleagues [24]. The observational learning variant was first described by Bellebaum and colleagues [20]. For both tasks, recording of participants’ responses and stimulus timing was controlled by Presentation Software (Neurobehavioral Systems Inc.; http://www.neurobs.com).

#### Active learning from feedback

**Figure 1:**
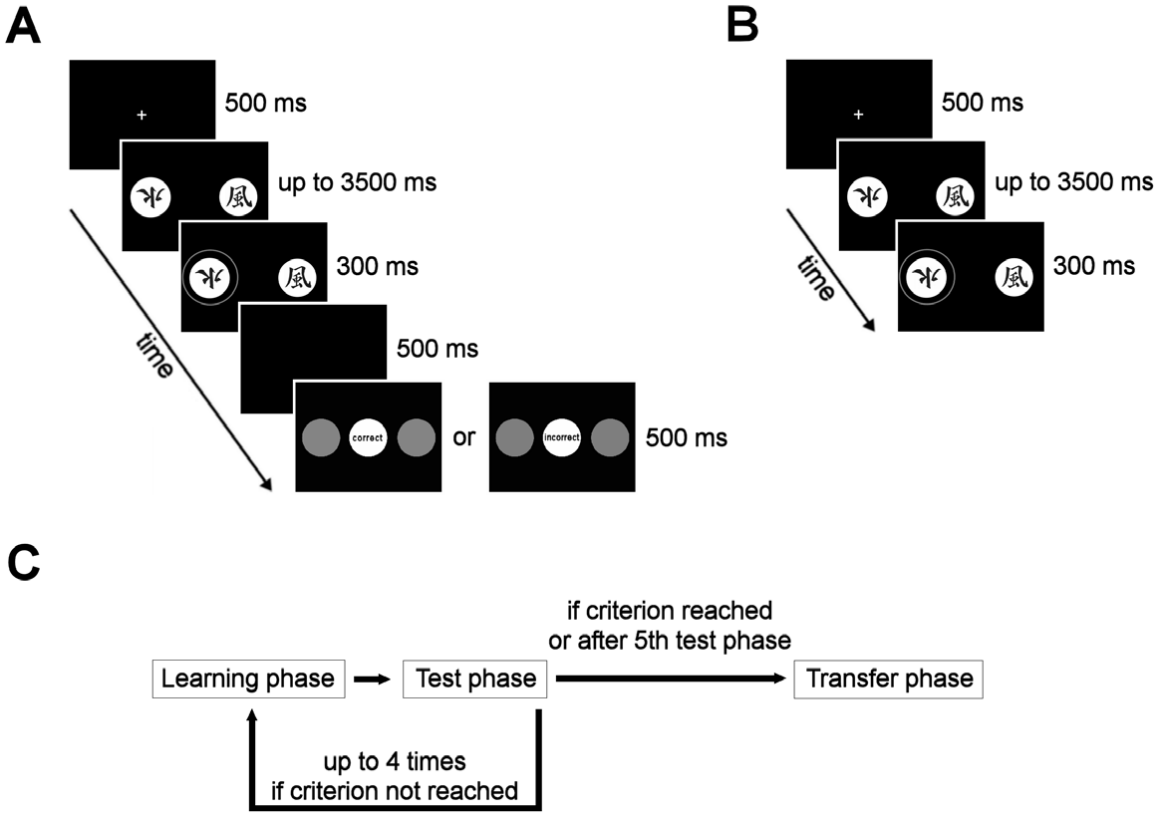
The active learning task. A) Time course of events in a single active learning trial. Subjects were presented a symbol pair and had to choose one of the symbols within 3500 ms via button press. Shortly after indication of the choice, positive (“correct”, grey circles appeared in green) or negative (“incorrect”, grey circles appeared in red) feedback was given. Subjects were instructed to pay attention to stimuli and associated outcomes in order to maximise the number of correct choices in all phases of the experiment. B) Time course of events in a single test or transfer phase trial. Trials in the test or transfer phase were identical to active learning trials except that no feedback was given. C) Learning and test phases alternated until subjects showed evidence of learning stimulus-outcome contingencies (maximally five learning and test phases were conducted; see Active Learning from Feedback for details). The task ended with the transfer phase (see Active Learning from Feedback for details).

In the learning phases of the active learning task, one of three symbol pairs (consisting of symbols A/B, C/D, E/F) was randomly presented on each of 60 trials (20 trials per symbol pair). Participants then had to choose between both symbols by pressing the left or right button of a response board. If participants did not respond within 3500 ms, the trial was scored as a miss, and they were asked to respond faster. If the button press occurred in time, the chosen symbol was indicated by a surrounding red circle. Shortly after, participants received either negative (“incorrect”) or positive (“correct”) feedback for their choice. Figure 1A illustrates the time course of events in active learning trials. The feedback enabled participants to learn which symbols were followed more often by positive feedback (A: 80%, C: 70%, E: 60%) relative to the alternative symbols (B: 20%, D: 30%, F: 40%). Each learning phase was followed by a test phase, in which participants were presented the same stimulus pairs, but their choices were not followed by feedback. Thus, subjects had to apply the knowledge they acquired during the learning phases. Test phases served to assess learning of stimulus-outcome contingencies. Each test phase consisted of 30 trials (10 per symbol pair; see Figure 1B for the time course of events in test trials). Previous studies have shown that the A/B pair is easiest to learn because of the highest reward probability for stimulus A and the lowest reward probability for stimulus B [19], [20]. However, learning to prefer stimulus A over B can result from learning that A leads to positive feedback, or from learning that B is associated with negative feedback, or from (a combination of) both. In order to disentangle the contributions of learning from positive and negative feedback, subjects completed a transfer phase, which followed after participants had reached at least eight choices of A and seven choices of C in any test phase (latest after five test phases; see Figure 1C for the course of learning, test and transfer phases). In the transfer phase, symbols A and B were paired with all other symbols, yielding the combinations A/C, A/D, A/E and A/F for stimulus A and the corresponding combinations for stimulus B, all of which were presented 5 times, resulting in 40 trials in total. As in the test phases, participants did not receive feedback for their choices in the transfer phase. A higher percentage of A choices (in the pairs involving stimulus A) relative to B avoidances (in the pairs involving stimulus B) is regarded as a positive learning bias, whereas the opposite pattern reflects a negative learning bias (“positive learning” vs. “negative learning”).

#### Observational learning from feedback

**Figure 2:**
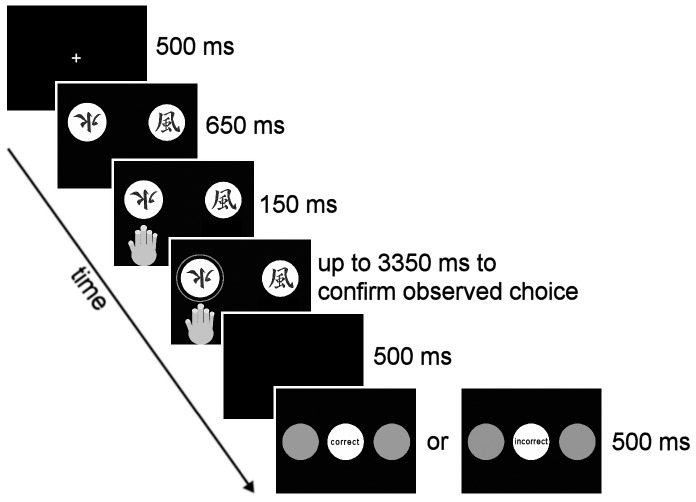
Time course of events in a single observational learning trial. After a symbol pair had been presented, subjects observed the choice of the virtual person. The choice had to be confirmed within 3350 ms in order to see feedback to the choice. As in the active learning task, subjects had to focus on choice-outcome associations in order to show correct responses in the trials of the test and transfer phases, which were identical to the active learning task.

The observational learning task was developed by Bellebaum and colleagues [20] to match the active learning task as closely as possible. Participants were asked to observe the performance of another person, introduced by name and a picture showing a sex- and approximately age-matched person on the computer screen. The observational learning task differed from the active learning task only with respect to the learning phase: In the observational version, the participants did not choose between the different stimuli themselves but observed the choices of another person. Each observed choice was indicated by the picture of a hand below the chosen symbol and a subsequently appearing red circle surrounding the symbol. In order to ensure attention to the task and to see the feedback the observed person received, participants had to confirm the observed choice by pressing the corresponding button (using the left or right button of the response board) within 3350 ms. Thus, participants in the observational version learned stimulus-outcome contingencies via feedback to observed choices as opposed to feedback to own choices in the active version of the learning task. Figure 2 shows the time course of events in observational learning trials. The test and transfer phases were identical for both the active and the observational learning task.

Unknown to the subjects, the observed person was only virtual: All observed responses were predetermined and balanced, i.e. all symbols (A–F) were chosen equally often. This was particularly important to not differentially promote learning from positive or negative feedback, as e.g. more frequent choices of A than B in the learning phase would have given subjects more often the opportunity to learn about stimulus A than learn about stimulus B.

### Testing Procedure

The main aim of the study was to examine the effect of altered DA levels on active and observational learning. Therefore, all patients were examined in the morning after overnight withdrawal of antiparkinsonian medication for at least 12 hours (OFF-state). Before testing, the Unified Parkinson’s Disease Rating Scale (UPDRS) [31] was determined (for UPDRS scores, see Table 1). Subsequently, each patient completed the (active or observational) feedback learning task. Control participants also performed the active or observational learning task.

Prior to participation, subjects were informed that the study purpose was the investigation of brain mechanisms of learning from feedback. After written consent had been given, the experiment was started.

### Statistical Design and Analysis

Data from both learning tasks (active and observational) were analysed separately in the first step. For both tasks, general feedback learning abilities were assessed by 1) the number of correct responses in the first test phase and 2) the number of test phases needed to reach a learning criterion. Similar to previous studies applying the probabilistic selection task [19], [24], the learning criterion was set to 70% correct choices for symbol pair A/B. All subjects reached this criterion and were included in the analysis of transfer phase performance. Finally, transfer phase performance was also compared between active and observational learning entering the data from both tasks into one statistical analysis.

PASW Statistics 18 (SPSS Inc.; http://www.ibm.com/software/analytics/spss) was used for statistical analyses. Data were analysed by means of mixed ANOVAs involving both within- and between-subjects factors, or with *t* tests, where appropriate (see Results section for more details). The level of significance was set to *p*<.05 (two-tailed) for all statistical analyses. When the sphericity assumption was violated, the Greenhouse-Geisser correction to adjust the degrees of freedom was applied. To resolve interactions, post-hoc repeated-measures ANOVAs and *t* tests (two-tailed) were performed, whenever necessary.

## Results

### Feedback Learning Tasks

#### Active learning from feedback

**Figure 3:**
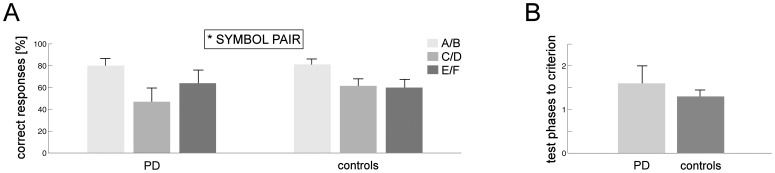
Test phase performance in the active learning task. A) Percentage of correct responses in the first test phase for symbol pairs A/B, C/D and E/F in unmedicated PD patients and controls. B) Number of test phases needed to reach the learning criterion (see Statistical Design and Analysis for details) in unmedicated PD patients and controls. Error bars represent standard errors (*SE*) of the mean; *p<.05.

Mean percentages of correct responses in the first test phase for PD patients and healthy controls are shown in Figure 3A. ANOVA with factors SYMBOL PAIR and GROUP yielded a significant main effect of SYMBOL PAIR (*F*_[2, 56]_ = 5.354; *p* = .012), with more correct responses for pair A/B than for C/D (*t*(29) = 3.926; *p*<.001) and E/F (*t*(29) = 2.587; *p* = .015). No performance difference was found for the comparison between C/D and E/F (*p*>.61). Neither the main effect of GROUP nor the GROUP×SYMBOL PAIR interaction reached significance (both *p*>.48).

Separately for both groups, Figure 3B shows the mean number of test phases needed to reach the learning criterion of at least 70% correct responses for pair A/B. No difference between groups was found (*p*>.39). Overall, learning success was thus comparable in patients and controls.

**Figure 4:**
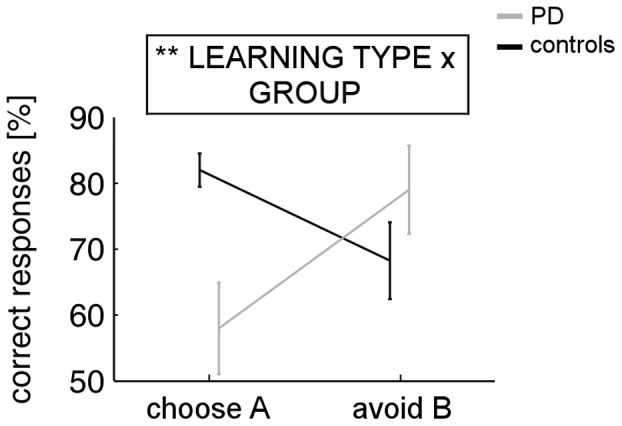
Transfer phase performance of controls and unmedicated PD patients in the active learning task. Percentages for ‘choose A’ and ‘avoid B’ indicate learning from positive and negative feedback, respectively. Error bars represent *SE*s; **p<.01.

Learning from positive and negative feedback as represented by transfer phase performance (‘choose A’ and ‘avoid B’, respectively), is shown for both groups in Figure 4. In the ANOVA with factors LEARNING TYPE and GROUP, none of the main effects reached significance (both *p*>.27), but a significant interaction between both factors was found (*F*[_1,28_] = 10.616; *p* = .003). A resolution of the interaction via paired-sample *t* tests showed that controls chose A significantly more often than they avoided B(*t*(19) = 2.307; *p* = .032), whereas the reverse pattern was found for PD patients, who avoided B more often than they chose A(*t*(9) = 2.264; *p* = .05). An alternative resolution of the interaction via independent-samples *t* tests reveals that this effect is driven more by between-group differences regarding choices of A(*t*(28) = 3.242; *p* = .007) than avoidances of B(*t*(28) = 1.13; *p* = .268).

#### Observational learning from feedback

**Figure 5:**
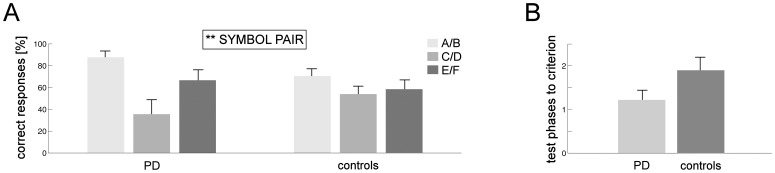
Test phase performance in the observational learning task. A) Percentage of correct responses in the first test phase for symbol pairs A/B, C/D and E/F in unmedicated PD patients and controls. B) Number of test phases needed to reach the learning criterion (see Statistical Design and Analysis for details) in unmedicated PD patients and controls. Error bars represent *SE*s; **p<.01.

As outlined in the Materials and Methods section, all subjects observed chance performance in the learning phases of the observational learning task. However, as in the active learning task, learning was assessed in test phases which involved responding without feedback. Figure 5A shows mean percentages of patients’ and controls’ correct responses in the first test phase. Similar as for active learning, ANOVA with factors SYMBOL PAIR and GROUP revealed a main effect of SYMBOL PAIR (*F*_[2,54]_ = 6.795; *p* = .002): Performance for A/B was significantly better than for C/D(*t*(28) = 3.576; *p* = .001) and tended to be better than for E/F (*t*(28) = 1.82; *p* = .08). No significant difference emerged for performance differences between C/D and E/F (*p*>.22). Neither the main effect of GROUP nor the GROUP × SYMBOL PAIR interaction reached significance (both *p*>.14).

The mean numbers of test phases needed to reach the learning criterion are depicted in Figure 5B. No difference between both groups was found (*p*>.16).

**Figure 6:**
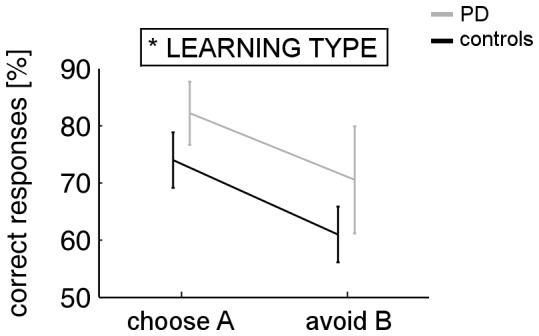
Transfer phase performance of unmedicated PD patients and controls in the observational learning task. Percentages for ‘choose A’ and ‘avoid B’ indicate learning from positive and negative feedback, respectively. Error bars represent *SE*s; *p<.05.

Scores for positive and negative feedback learning – as assessed by the transfer phase of the observational learning task – are illustrated for both groups in Figure 6. In the ANOVA with factors LEARNING TYPE and GROUP, a significant main effect of LEARNING TYPE (*F* [_1]_, [27] = 4.558; *p* = .042) indicates better learning from positive than negative feedback across both groups. The main effect of GROUP did not reach significance, as did the LEARNING TYPE × GROUP interaction (both *p*>.2).

### Comparison of Transfer and Test Phase Performance between Active and Observational Learning

Repeated-measures ANOVA with within-subjects factor LEARNING TYPE (positive vs. negative) and the between-subjects factors LEARNING TASK (active vs. observational) and GROUP (PD vs. healthy controls) yielded a significant three-way interaction (*F*_[1, 55]_ = 4.527; *p* = .038). A resolution of the interaction via independent-samples *t* tests revealed that PD patients learned better from positive feedback in the observational as compared to the active learning task (*t*(17) = 2.683; *p* = .016), whereas no difference was found for negative feedback (*p*>.46). In healthy controls, however, differences between active and observational learning were found neither for positive nor negative feedback (both *p*>.15). In addition, a significant LEARNING TYPE × LEARNING TASK interaction emerged (*F*_ [1, 55]_ = 4.130; *p* = .047), indicating better learning from positive feedback in the observational (*t*(28) = 2.397; *p* = .023) but not in the active learning task (*p*>.70). According to a resolution of a significant LEARNING TYPE × GROUP interaction (*F*_[1, 56]_ = 5.923; *p* = .018), overall better learning from positive as compared to negative feedback was found in healthy controls (*t*(39) = 3.384; *p* = .002) but not in PD patients (*p* = .52). All remaining effects in the ANOVA did not reach significance (*p*>.27).

Although the numbers of test phases needed to reach the learning criterion did not differ between PD and healthy controls (see Results separately for active and observational learning from feedback), visual inspection of Figures 3B and 5B suggested the possibility of a significant LEARNING TASK by GROUP interaction. Univariate ANOVA, however, yielded only a trend for this interaction (*F*_[1, 55]_ = 2.819; *p* = .099) with neither of the main effects reaching significance (both *p*>.51).

## Discussion

In the present study, PD patients and healthy controls completed either an active or an observational learning variant of a probabilistic selection task [24]. In line with a recent study by Shiner and colleagues [32], who showed learning of stimulus-outcome contingencies independent from dopaminergic drug state (ON vs. OFF) in PD patients, learning of contingencies was comparable between unmedicated PD patients and healthy controls for both the observational and the active learning task in the present study. A different pattern, however, emerged for learning from positive and negative feedback, which was assessed separately in both variants of the task. As hypothesized, a bias to learn better from negative feedback was only present in actively learning PD patients OFF medication, whereas patients who learned by observation showed the same behavioural pattern as healthy controls, who learned better from positive feedback across tasks.

Importantly, learning histories differed in the active and observational learning task at hand, i.e. observational learners observed chance performance. This procedure was chosen because we aimed to give both active and observational learners the chance to learn from positive and negative feedback. Although learning histories differed in both tasks, we consider it unlikely that these differences can explain the present findings. Different learning biases for active and observational learning were seen in PD patients, whereas for healthy controls differences between active and observational learning were found neither for positive nor for negative feedback. If, for example, the observation of chance performance led to enhanced positive learning, this effect would also be expected to occur in control subjects. Nevertheless, we further explored a potential relationship between learning history and learning bias in actively learning healthy controls, because also in active learning, subjects may by chance choose the better option more often from the beginning, which would impair their ability to learn about the alternative option. The correlation between the number of correct responses for the A/B pair during the learning phase and the learning bias, i.e. the difference between positive and negative feedback learning in the transfer phase, was far from reaching significance. At least for active learning, different learning histories do thus not affect the learning bias. Albeit we cannot completely exclude the possibility of an interaction between learning history and learning task (active vs. observational), such an interaction is unlikely, because, as outlined above, controls showed comparable learning biases in active and observational learning.

We are aware that observing a non-learning agent differs from most real-life situations of observational learning. It is conceivable that observing an intelligent agent who performs above chance leads to more successful subsequent behaviour than observing chance performance. It has to be noted, however, that active behaviour following the observation of a very good performer can reflect both learning and imitation processes, which cannot be disentangled. This was a further motivation for us to let subjects observe chance performance.

Together, the present findings strongly suggest that DA level reductions affect active learning more than observational learning. It has been suggested that improved active learning from negative feedback in PD patients OFF medication is caused by DA depletion, because the reverse pattern, i.e. superior learning from positive feedback, was found with dopamine medication [24]. Frank and colleagues [24] explained this finding by means of their computational model of basal ganglia-dopamine interactions in cognition. According to this model, the striatum contains Go and NoGo cells, both of which are differentially affected by DA. Reduced DA activity – as in unmedicated PD patients – leads to less excitation of Go cells via D1 receptors and stronger activation of NoGo cells via D2 receptors. Consequently, the Go signal, which facilitates action execution, is diminished, whereas the NoGo signal, which is thought to suppress action execution, is enhanced. Therefore, unmedicated PD patients are better at learning to suppress actions – as promoted by negative feedback – than at learning to execute actions – as promoted by positive feedback [24].

The present study suggests that this model applies only to actions which are actively executed, but not to actions which are merely observed by the learner, as indicated by comparable observational learning in unmedicated PD patients relative to healthy controls in the present study. More specifically, the inhibition of action selection caused by DA depletions, which underlies the negative learning bias in active learning, does not lead to a negative learning bias in observational learning, because negative outcomes in observational learning are not related to own action.

Most likely, this dissociation in learning tendencies is caused by a differential recruitment of neural structures of the reward system in active and observational feedback learning. Functional neuroimaging studies on the role of the striatum in feedback-based learning revealed prediction-error related modulation of activity for the midbrain and the ventral [33], [34] as well as dorsal striatum [11] when outcomes followed one’s own choices (for a review on neural substrates of reward prediction, see [12]). On the contrary, a modulation was found only for the ventral but not dorsal striatum in case of outcomes which were not related to actions [11] as well as during observational learning [16]. These findings support the suggestion that each part of the striatum is involved in a distinct function of reinforcement learning, with the dorsal part playing a crucial role in learning of action-outcome contingencies (“actor”) and the ventral part contributing to the learning of stimulus-outcome contingencies (“critic”) [11]. Consequently, observational learning, in which neither action facilitation nor suppression is required during learning, may depend less on the dorsal striatum. Presumably, this is one reason why reduced DA input to the striatum does not cause a bias towards enhanced action suppression in unmedicated PD patients learning by observation. At the same time, DA depletion may be less severe in the ventral than in the dorsal striatum in PD patients [26]. MacDonald and colleagues [35] showed larger effects of dopamine replacement on functions related to the dorsal striatum as compared to those related to the ventral striatum in PD [35]. Interestingly, a recent fMRI study by our group revealed stronger prediction error related activations in a small part of the anterior caudate nucleus for active compared to observational learning [21], resembling the reported activations seen for instrumental vs. classical conditioning [11]. Thus, part of the striatum appears to be responsible for linking (own) actions and outcomes.

At first sight, it may be surprising that healthy controls in the active learning task and both controls and patients in the observational learning task learned better from positive than negative feedback, because an opposite bias was reported for older seniors (mean age 77 years) [36], and no bias for younger seniors below 70 years of age [36], [37]. In a group of participants with a mean age of 60 years, Bellebaum and colleagues found no bias for active learning either. For observational learning, however, better learning from positive feedback was reported for another group of comparable age [20]. These findings were explained by the so-called ‘positivity effect’, which refers to preserved positive but – up to the age of about 60– continuously reduced negative affect in healthy aging (see [38]). In line with this effect, participants in the present study learned better from positive than negative feedback when DA levels were normal, i.e. in healthy controls, or when reduced DA levels had no effect on learning, i.e. in unmedicated PD patients learning by observation. Interestingly, our result of healthy subjects’ general bias in favour of positive feedback was not found for active learning by Bellebaum and colleagues [20]. Note, however, that healthy subjects in our study were on average about five years younger. It is possible that a bias to learn better from positive feedback (presumably caused by the positivity effect) is counterbalanced at the age of 60 to 70 by alterations in the neural structures of the reward system which accompany healthy aging [39]–[41]. While the positivity effect is assumed to remain stable after the age of about 60, further changes of the reward system may promote negative learning and thus balance the relative tendency in learning, whereas the positivity effect may be predominant in observational learning due to diminished involvement of the reward system.

In the present study, the negative learning bias in PD patients is caused more by reduced positive learning than by increased negative learning compared to healthy controls, whereas Frank and colleagues reported elevated negative learning performance in PD patients [24]. This result pattern may be related to slight differences in the study sample and in the experimental procedures. Subjects in the study by Frank and colleagues were on average about 8 years older than in the present study, so that age-related changes (see above) may have added to PD-related changes of the reward system, presumably leading to better negative learning. Furthermore, Frank and colleagues excluded those subjects who were generally confused by not receiving feedback for their choices in the transfer phase, whereas in our study learning was specifically assessed in test trials without feedback.

A similarity between the present study and the study by Frank and colleagues [24] is that patients were not excluded based on depressive symptoms. Importantly, different results regarding patients learning actively and by observation cannot be explained by increased depression scores in the patients, as the scores were comparable between both groups.

In conclusion, the present study shows a dissociation between effects of DA level reductions on active and observational feedback learning. Reduced DA levels result in improved active learning from negative feedback, whereas superior learning from positive feedback was found in observational learning, both for unmedicated PD patients and healthy controls. Phasic DA bursts and dips following rewarding or punishing stimuli facilitate or inhibit action selection, which directly leads to positive and negative learning when active choices are required. Accordingly, changes in the DA level alter the relative tendency for positive and negative learning, with DA depletion promoting negative learning. The same associations between stimuli and actions can, however, be learned by observation, without significant effects of DA level reductions. This finding sheds new light on the mechanisms involved in feedback processing and feedback learning.
